# Socioeconomic status correlates with clinical outcomes in patients with acral melanoma

**DOI:** 10.3389/fpubh.2025.1496082

**Published:** 2025-02-03

**Authors:** Rong Huang, Jiayu Wang, Kelin Zheng, Mengke Zhao, Zhengyun Zou

**Affiliations:** ^1^Department of the Comprehensive Cancer Center, Nanjing Drum Tower Hospital, Affiliated Hospital of Medical School, Nanjing University, Nanjing, China; ^2^Nanjing Drum Tower Hospital Clinical College of Nanjing University of Chinese Medicine, Nanjing, China; ^3^Nanjing Drum Tower Hospital Clinical College of Nanjing Medical University, Nanjing, China

**Keywords:** acral melanoma, socioeconomic status, Breslow thickness, survival, prognostic factors

## Abstract

**Objectives:**

This study aimed to investigate the impact of socioeconomic status on survival in Chinese patients with acral melanoma.

**Methods:**

We collected clinical and socioeconomic information of 298 primary acral melanoma patients and performed Kaplan–Meier curves, log-rank tests, Cox proportional hazards models, and Pearson’s chi-squared tests to evaluate the relationships between clinical characteristics, socioeconomic factors, and survival outcomes.

**Results:**

Among the clinical characteristics, age, gender, stage, Breslow thickness, and primary tumor site significantly impacted survival in acral melanoma patients (*p* = 0.01, *p* = 0.05, *p* < 0.01, *p* < 0.01, *p* < 0.01, respectively). Compared to individual socioeconomic factors such as education, occupation, medical insurance, and marital status, the socioeconomic level derived from these four dimensions accurately predicted patient survival. Patients with higher socioeconomic status demonstrated significantly reduced mortality risk (HR = 0.60, 95% CI = 0.40–0.91, *p* = 0.02). However, socioeconomic level was not found to be an independent prognostic factor for acral melanoma patients after multivariable adjustment. Notably, a negative correlation was observed between socioeconomic level and Breslow thickness.

**Conclusion:**

Socioeconomic level is associated with survival in Chinese acral melanoma patients. However, this association may be attributable to Breslow thickness rather than socioeconomic status itself.

## Introduction

Melanoma is a melanocyte-derived malignant tumor with a generally poor prognosis. Currently, its prevalence is on the rise worldwide. From 1990 to 2019, the incidence of melanoma has surged by 170%, while deaths have increased by 90% globally (1), which is accompanied by a huge treatment-related financial burden. The 5-year overall survival (OS) rate for Chinese melanoma patients stands at 41.6%, and the median OS is 3.92 years, with the 5-year OS rate of merely 4.6% for stage IV patients (2). Acral melanoma (AM), which constitutes nearly half of Chinese melanoma cases, primarily affects palmar, plantar, and subungual skin (2, 3). Characterized by its insidious onset and advanced stage at diagnosis (4), AM is associated with an unfavorable prognosis compared to non-acral cutaneous melanoma (5, 6). In our previous retrospective study, the estimated median OS for AM patients undergoing surgery was 60 months (7).

Previous studies have investigated the link between melanoma and socioeconomic status (SES), revealing that high SES correlates with increased melanoma incidence, thinner tumor thickness, and prolonged survival (7–22). These studies have used diverse assessment measures to evaluate SES. The most commonly used SES factors impacting melanoma include educational attainment, income, occupation, medical insurance, and marital status. Evaluation indicators vary across different studies and have certain limitations, often reflecting specific aspects of SES rather than comprehensively capturing its entirety. For example, some studies have analyzed the effect of single SES factors on melanoma survival, such as educational level (16, 22), occupation (17), income (18, 19), or marital status (20, 23). Unsurprisingly, other studies evaluate SES using multiple indicators (8, 10–14, 21). Considering that different SES indicators may measure different dimensions of SES, it is necessary to comprehensively study multiple SES dimensions.

However, to date, no study has confirmed the prognostic value of SES in Chinese AM patients. Inequalities in access to treatment and care exist even within a universal healthcare system (9). Within the context of a non-universal healthcare system in China, it is particularly important to analyze the relationship between SES level, including medical insurance status, and the prognosis of AM patients.

For this purpose, we assessed the SES levels of Chinese AM patients across four dimensions in this study: educational attainment, occupation, medical insurance, and marital status. Through a multi-dimensional analysis of SES and clinical features, we identified a significant association between SES and survival. Additionally, SES was negatively correlated with Breslow thickness. Our findings seek to furnish evidence-based insights for health departments to formulate health promotion strategies and public health policies.

## Methods

### Patient population and data collection

A total of 298 consecutive primary AM patients were collected from July 2010 to November 2021 at Drum Tower Hospital after excluding those with incomplete SES data. All patients enrolled in this study had been diagnosed by histopathology as having melanoma found on acral skin, including any histopathological subtypes such as acral lentiginous melanoma, nodular melanoma, and superficial spreading melanoma. The clinical and socioeconomic data were collected, including age, gender, stage at initial diagnosis (AJCC 8th Edition), primary tumor site, educational attainment, occupation, medical insurance, marital status, and survival status until the last follow-up or death.

Income data were not collected due to concerns about privacy, variability, and difficulty of accurate quantification, particularly for farmers. This retrospective study received approval from the Ethics Committee of Drum Tower Hospital (2024–277). Informed consent was waived due to the study’s retrospective nature and the anonymization of patient data.

### Assessment of SES level

This study categorized educational attainment among AM patients as illiteracy, nine-year compulsory education, high school, or university/college. Occupations were classified as unemployment, farmers or manual laborers, or other non-manual professions. In addition, the status of medical insurance in AM patients included uninsured status, rural or urban residents’ medical insurance, and staff medical insurance. As rural and urban residents’ medical insurance share similarities and act as the basic medical insurance system for the unemployed in China (24), they were combined into a single category termed primary medical insurance. Marital status was categorized as married and other status, including never married, divorced, and widowed.

Each indicator was assigned different weight scores based on their degrees, and detailed rules were shown in Supplementary Table S1. The sum of the scores of each indicator constituted the SES score. The optimal cut-off value of the SES score was determined using X-tile bioinformatics software (Version 3.6.1). Higher scores represented better SES levels. Patients scoring between 3 and 7 were categorized as high SES, while scores of 0 to 2 denoted low SES.

### Statistical analysis

The Kaplan–Meier estimate was used to analyze the survival of various patient groups and median OS. The median follow-up time was determined according to the reverse Kaplan–Meier method. Log-rank tests and Cox proportional hazards models were utilized to identify prognostic factors and assess the risk of death for various covariates. Pearson’s chi-squared test was used for correlation analysis of categorical variables. A two-sided *p-value* of no more than 0.05 was considered statistically significant (*p ≤ 0.05). The R Project for Statistical Computing (version 4.3.3) and IBM SPSS Statistics (version 25.0) were used to perform these statistical analyses.

## Results

### Patient characteristics

A total of 298 primary AM patients were included in this study. The median age of initial diagnosis was 60 years old (range: 22–87 years old). Among them, 135 (45.3%) patients were female, and 163 (54.7%) were male. Most patients presented with stage II (39.3%) or stage III (29.2%) disease, with Breslow thickness > 2.0 mm observed in the majority (54.0%) of cases. Primary lesions were predominantly located in the feet (84.6%). The median follow-up time was 36 months (4–128 months). At the last follow-up, 90 patients had died. And the estimated median OS was 60.0 months.

The AM patients in this study had a relatively low level of education, and 71.1% of patients only received or did not receive 9 years of compulsory education. Over half of the patients were farmers or manual laborers, and 35.9% were engaged in non-manual professions such as teachers, civil servants, and white-collar. In addition, 16 patients were unemployed. 30.5% of patients did not participate in any medical insurance, 26.8% of patients participated in basic medical insurance (rural or urban residents’ medical insurance), and 42.6% of patients purchased staff medical insurance. Most patients were married (95.3%), while 4.7% were in other marital status (including 2 never married, 2 divorced, and 10 widowed). The clinical and demographic characteristics are summarized in Table 1.

**Table 1:** Clinical and demographic characteristics of all patients.

Variable, *n* (%)	Low SES	High SES	Total
Age (years)			
≥ 60	61 (40.4%)	90 (59.6%)	151
< 60	40 (27.2%)	107 (72.8%)	147
Gender			
Women	57 (42.2%)	78 (58.5%)	135
Men	44 (27.0%)	119 (73.0%)	163
Stage			
0	3 (27.3%)	8 (72.7%)	11
I	5 (16.1%)	26 (83.9%)	31
II	42 (35.9%)	75 (64.1%)	117
III	32 (36.8%)	55 (63.2%)	87
IV	9 (47.4%)	10 (52.6%)	19
Missing	10 (30.3%)	23 (69.7%)	33
Breslow thickness (mm)			
≤ 1.0	7 (21.9%)	25 (78.1%)	32
1.01–2.0	12 (24.0%)	38 (76.0%)	50
2.01–4.0	27 (38.0%)	44 (62.0%)	71
> 4.0	37 (41.1%)	53 (58.9%)	90
Missing	18 (32.7%)	37 (67.3%)	55
Primary site			
Hand	11 (23.9%)	35 (76.1%)	46
Foot	90 (35.7%)	162 (64.3%)	252
Medical insurance			
No insurance	72 (79.1%)	19 (20.9%)	91
Rural or urban residents’ medical insurance	29 (36.3%)	51 (63.7%)	80
Staff medical insurance	0 (0.0%)	127 (100.0%)	127
Educational attainment			
Illiteracy	53 (82.8%)	11 (17.2%)	64
Compulsory education	48 (32.4%)	100 (67.6%)	148
Senior high school	0 (0.0%)	58 (100.0%)	58
University/College	0 (0.0%)	28 (100.0%)	28
Occupation			
Unemployment	15 (93.8%)	1 (6.3%)	16
Farmer/Manual laborers	86 (49.1%)	89 (50.9%)	175
Other non-manual professions	0 (0.0%)	107 (100.0%)	107
Marital status			
Married	92 (32.4%)	192 (67.6%)	284
Other status	9 (64.3%)	5 (35.7%)	14
Survival			
Alive	60 (28.8%)	148 (71.2%)	208
Dead	38 (42.2%)	52 (57.8%)	90

### Univariate survival analysis

Among these clinical and demographic characteristics, age, gender, stage, Breslow thickness, and primary site were significantly associated with AM patient survival (*p* < 0.01, *p* = 0.05, *p* < 0.01, *p* < 0.01, *p* < 0.01, respectively; Table 2). Educational attainment, occupation, and marital status did not significantly affect patient survival. However, the univariate COX analysis showed that medical insurance status was significantly associated with survival. Patients with staff medical insurance had longer survival times than those without any insurance (hazard ratio (HR) = 0.60, 95% confidence interval (CI) = 0.37–0.96, *p* = 0.03). Additionally, log-rank tests for individual socioeconomic factors did not confirm their prognostic significance (Supplementary Figure S1).

**Table 2:** Univariate and multivariate analyses of factors associated with overall survival.

Factor	Variable	Univariate analysis		Multivariate analysis	
		HR (95% CI)	*p* value	HR (95% CI)	*p* value
Age	≥60 vs. <60	1.76 (1.15–2.69)	<0.01	1.91 (1.10–3.32)	0.02
Gender	Men vs. women	1.53 (1.00–2.35)	0.05	1.62 (0.92–2.86)	0.10
Stage	III-IV vs. 0-II	3.67 (2.26–5.96)	<0.01	3.57 (2.11–6.06)	<0.01
Breslow thickness	>2 vs. ≤2	3.37 (1.71–6.64)	<0.01	3.02 (1.49–6.11)	<0.01
Primary site	Foot vs. hand	3.24 (1.41–7.43)	<0.01	5.07 (1.21–21.30)	0.03
Medical insurance	No insurance				
	Basic medical insurance	0.78 (0.46–1.33)	0.37	1.01 (0.46–2.21)	0.98
	Staff medical insurance	0.60 (0.37–0.96)	0.03	0.98 (0.40–2.41)	0.96
Educational attainment	Illiteracy				
	Compulsory education	0.84 (0.50–1.42)	0.52		
	Senior high school	0.85 (0.45–1.62)	0.63		
	University/college	0.80 (0.35–1.80)	0.59		
Occupation	Unemployment				
	Farmer/manual laborers	0.68 (0.31–1.51)	0.34		
	Other non-manual professions	0.67 (0.30–1.52)	0.34		
Marital status	Married vs. other status	0.49 (0.21–1.12)	0.09		
SES level	High vs. low	0.60 (0.40–0.91)	0.02	0.64 (0.29–1.44)	0.28

Basic medical insurance represented rural and urban residents’ medical insurance. Other statuses included never married, widowed, and divorced.

**Figure 1:**
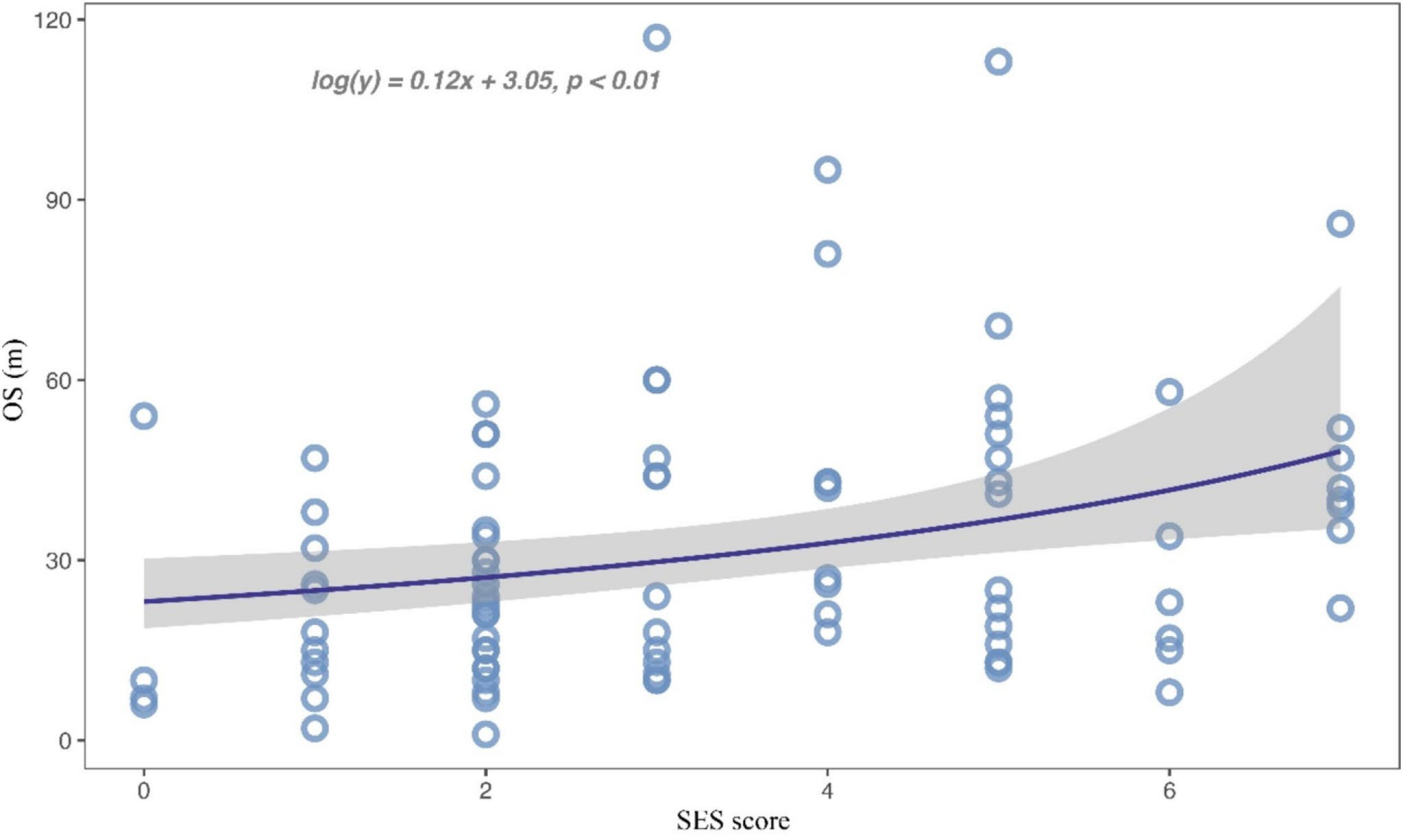
Scatterplot for the relationship between SES score and overall survival time.

**Figure 2:**
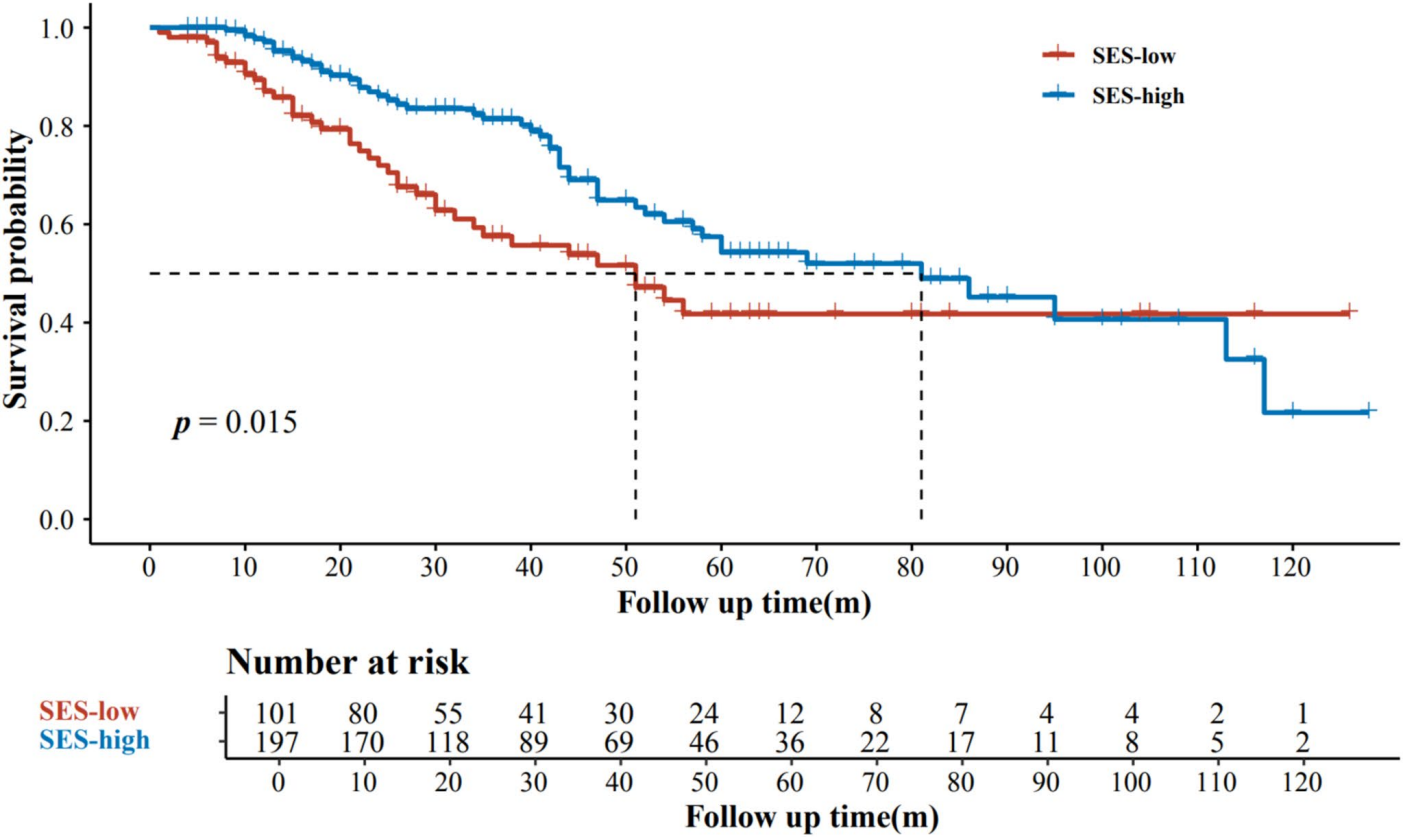
Kaplan–Meier estimates of overall survival according to SES levels.

Subsequently, we combined educational attainment, occupation, medical insurance, and marital status to obtain a SES score for each patient. Detailed scoring rules are shown in Supplementary Table S1. And the SES score showed a positive association with survival (Figure 1). Patients with higher SES scores exhibited longer survival times. Additionally, patients were categorized into two groups according to their comprehensive SES scores. We further analyzed the difference in OS between patients with high and low SES scores through the log-rank test (*p* = 0.015; Figure 2). It was found that the SES could predict patient survival more precisely than any single socioeconomic factor (Figures 2; Supplementary Figure S1). Patients with high SES had significantly reduced AM mortality risk (HR = 0.61, 95% CI = 0.40–0.92, *p* = 0.02; Table 2).

### Multivariate survival analysis

**Figure 3:**
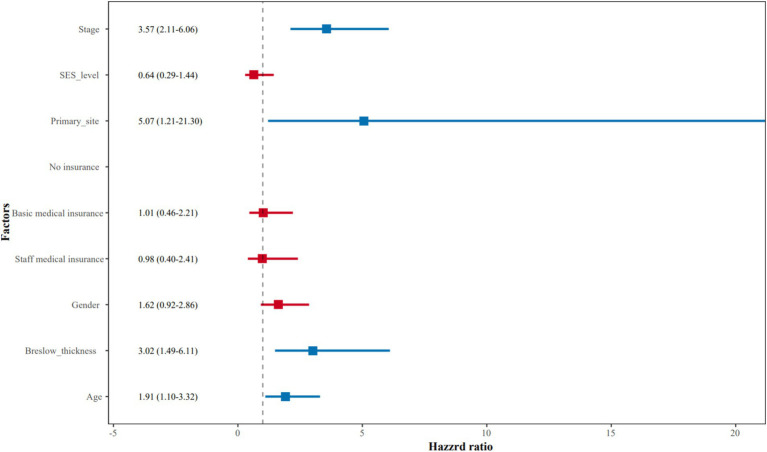
The forest plot of multivariate Cox regression analysis for overall survival.

After adjustment for prognostic factors such as age, gender, primary site, Breslow thickness, and stage at initial diagnosis, SES level (HR = 0.64, 95% CI = 0.29–1.44, *p* = 0.28) and staff medical insurance (HR = 0.98, 95% CI = 0.40–2.41, *p* = 0.96) were not independent predictors of AM patient prognosis. Patients with advanced stages (stage III and stage IV) had shorter OS (HR = 3.57, 95% CI =2.11–6.06, *p* < 0.01) compared to those with earlier stages (stage 0, stage I, and stage II). In addition, we found that AM patients with lesions located in the feet also showed a worse prognosis compared to those in the hands (HR = 5.07, 95% CI =1.21–21.30, *p* = 0.03), although with a wide 95% CI interval due to a small sample size of the hand subgroup. Patients with Breslow thickness > 2.0 mm had worse survival than those with Breslow thickness ≤ 2.0 mm (HR = 3.02, 95% CI 1.49–6.11, *p* < 0.01). Moreover, patients with older ages also had poorer survival compared with younger patients (HR = 1.91, 95% CI 1.10–3.32, *p* = 0.02). Multivariate Cox regression analysis for OS was summarized in Table 2 and presented as a forest plot (Figure 3).

### Associations between SES and clinical characteristics

**Figure 4:**
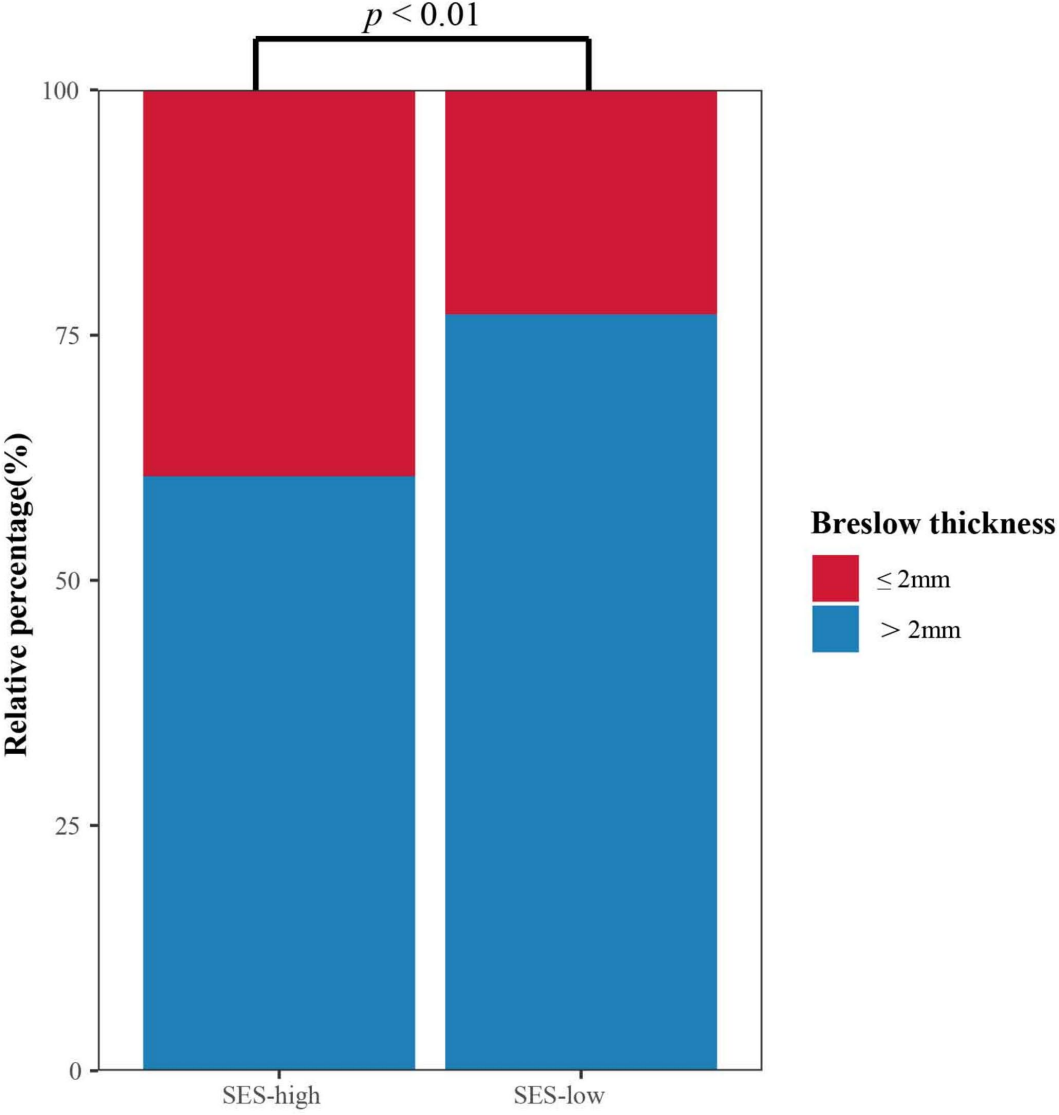
Stacked column chart of Breslow thickness in different SES groups.

Furthermore, the association analysis between SES and crucial‌ clinical characteristics was investigated. It was Breslow thickness rather than the stage at initial diagnosis that was significantly negatively associated with SES (Pearson’s chi-square = 6.64, *p* < 0.01; Pearson’s chi-square = 1.48, *p* = 0.22), as shown in Supplementary Table S2. Patients presenting with Breslow thickness ≤ 2 mm were more likely to have high SES. The stacked column chart revealed the distribution of Breslow thickness in high and low SES groups (Figure 4). Medical insurance, occupation, education, and marital status were neither associated with tumor thickness nor stage (Supplementary Table S3–S6).

## Discussion

Previous studies have established SES as a significant predictor of melanoma prognosis (11, 14, 15). In this study, we demonstrated the predictive role of SES in the prognosis of Chinese AM patients, finding that the combined SES level predicted survival more accurately than individual socioeconomic factors. However, SES did not act as an independent prognostic factor in AM. A negative correlation was observed between SES level and Breslow thickness, potentially explaining the better survival outcomes among patients with higher SES. To the best of our knowledge, this is the first Chinese population-based analysis evaluating the relationship between SES and AM survival and clinical characteristics.

Patients with a college education are generally more informed about melanoma (25). A stable job, medical insurance, and marriage help patients access timely anti-melanoma treatment and reduce disease-related economic burden (20, 26). A study has reported the high economic burden of melanoma patients with macroscopic lymph node metastasis or distant metastasis (27). Many patients in this study had low SES indicators, with a notable proportion being unemployed, illiterate, or medically uninsured. Single socioeconomic factors failed to serve as prognostic factors in log-rank tests. As a comprehensive measure of socioeconomic factors, SES score played a role in predicting patient survival, emphasizing the significance of evaluating SES comprehensively.

Although SES was not an independent prognostic factor in the multivariate analysis, its association with survival emphasizes its indirect impact through clinical factors such as Breslow thickness. Previous studies have consistently shown that low SES is linked to advanced stages of melanoma (15, 28, 29). The negative relationship between SES and Breslow thickness has also been revealed (8, 21). Indeed, AM often presents with thick tumors and advanced stages, especially for Asian patients (30), and these characteristics are associated with poorer survival (6, 31, 32). Our study corroborates these findings, demonstrating that patients with high SES were more likely to present with Breslow thickness ≤ 2.0 mm. This relationship likely explains the survival differences between high-and low-SES groups.

Our study has several limitations. First, this is a single-center study with a limited sample size. Second, our measures of SES were limited and may not have fully characterized an individual’s SES level. Despite these constraints, our findings suggest that AM patients with low SES levels have a higher mortality risk, which may be related to thicker tumor thickness. For AM, patients are commonly diagnosed at an advanced stage and with a thick tumor. Therefore, this study highlights that early diagnosis and treatment are crucial for AM patients, especially those with low SES levels.

## Data Availability

The raw data supporting the conclusions of this article will be made available by the authors, without undue reservation.
