# Supplementary material for: Socioeconomic status correlates with clinical outcomes in patients with acral melanoma
Source: Front Public Health. 2025 Feb 3;13:1496082. doi: 10.3389/fpubh.2025.1496082 (PMC11830742; doi:10.3389/fpubh.2025.1496082)
Supplement: Supplementary file 1 [file Table_1.docx]

Table S1. Score scale of SES.

| **Socioeconomic status** | **Category** | **Assessment score** |
| --- | --- | --- |
| Medical insurance | No insurance | 0 |
|  | Rural or urban residents’ medical insurance | 1 |
|  | Staff medical insurance | 2 |
| Educational attainment | Illiteracy | 0 |
|  | Compulsory education | 1 |
|  | Senior high school | 2 |
|  | University/College | 3 |
| Occupation | Unemployment | 0 |
|  | Farmer/ Manual laborers | 1 |
|  | Other non-manual professions | 2 |
| Marital status | Married | 0 |
|  | Other status (never married, widowed and divorced) | -1 |

Table S2. Constituent ratios of Breslow thickness and stage in different SES groups

| Factors | | Low SES | High SES | Total | chi-square test |
| --- | --- | --- | --- | --- | --- |
|  |  |  |  |  | **χ^2^ *p* value** |
| Breslow thickness | ≤2mm | 19^a^ (23.2%) | 63^b^ (76.8%) | 82 (100%) | 6.64 < 0.01 |
|  | ＞2mm | 64^a^ (39.8%) | 97^b^ (60.2%) | 161 (100%) |  |
|  | Total | 83 (34.2%) | 160 (65.8%) | 243 (100%) |  |
| Stage | 0-Ⅱ | 50^a^ (31.4%) | 109^a^ (68.6%) | 159 (100%) | 1.48 0.22 |
|  | Ⅲ-Ⅳ | 41^a^ (38.7%) | 65^a^ (61.3%) | 106 (100%) |  |
|  | Total | 91 (34.3%) | 174 (65.7%) | 265 (100%) |  |

Different superscripts between columns indicate statistical difference (*p* < 0.05).

Same-letter superscripts indicate no statistical difference (*p*＞0.05).

Table S3. Constituent ratios of Breslow thickness and stage in different medical insurance groups

| Factors | | No insurance | Basic medical insurance | Staff medical insurance | Total | chi-square test |
| --- | --- | --- | --- | --- | --- | --- |
|  |  |  |  |  |  | **χ^2^ *p* value** |
| Breslow thickness | ≤2mm | 18^a^ (22.0%) | 21^a^ (25.6%) | 43^a^ (52.4%) | 82 (100%) | 5.40 0.07 |
|  | ＞2mm | 56^a^ (34.8%) | 43^a^ (26.7%) | 62^a^ (38.5%) | 161 (100%) |  |
|  | Total | 74 (30.5%) | 64 (26.3%) | 105 (43.2%) | 243 (100%) |  |
| Stage | 0-Ⅱ | 46^a^ (28.9%) | 38^a^ (23.9%) | 75^a^ (47.2%) | 159 (100%) | 2.81 0.25 |
|  | Ⅲ-Ⅳ | 36^a^ (34.0%) | 31^a^ (29.2%) | 39^a^ (36.8%) | 106 (100%) |  |
|  | Total | 82 (30.9%) | 69 (26.0%) | 114 (43.0%) | 265 (100%) |  |

Same-letter superscripts indicate no statistical difference (*p*＞0.05).

Table S4. Constituent ratios of Breslow thickness and stage in different occupation groups

| Factors | | Unemployment | | Farmer/Manual laborers | | Other non-manual professions | Total | chi-square test |
| --- | --- | --- | --- | --- | --- | --- | --- | --- |
|  |  |  |  |  |  |  |  | **χ^2^ *p* value** |
| Breslow thickness | ≤2mm | | 6^a^ (7.3%) | | 46^a^ (56.1%) | 30^a^ (36.6%) | 82 (100%) | 0.13 0.94 |
|  | ＞2mm | | 10^a^ (6.2%) | | 93^a^ (57.8%) | 58^a^ (36.0%) | 161 (100%) |  |
|  | Total | | 16 (6.6%) | | 139 (57.2%) | 88 (36.2%) | 243 (100%) |  |
| Stage | 0-Ⅱ | | 10^a^ (6.3%) | | 91^a^ (57.2%) | 58^a^ (36.5%) | 159 (100%) | 0.07 0.97 |
|  | Ⅲ-Ⅳ | | 6^a^ (5.7%) | | 62^a^ (58.5%) | 38^a^ (35.8%) | 106 (100%) |  |
|  | Total | | 16 (6.0%) | | 153 (57.7%) | 96 (36.2%) | 265 (100%) |  |

Same-letter superscripts indicate no statistical difference (*p*＞0.05).

Table S5. Constituent ratios of Breslow thickness and stage in different education groups

| Factors | | | Illiteracy | Compulsory education | Senior high school | University/College | Total | chi-square test |
| --- | --- | --- | --- | --- | --- | --- | --- | --- |
|  |  |  |  |  |  |  |  | **χ^2^ *p* value** |
| Breslow thickness | ≤2mm | 15^a^ (18.3%) | | 39^a^ (47.6%) | 19^a^ (23.2%) | 9^a^ (11.0%) | 82 (100%) | 1.58 0.66 |
|  | ＞2mm | 38^a^ (23.6%) | | 79^a^ (49.1%) | 30^a^ (18.6%) | 14^a^ (8.7%) | 161 (100%) |  |
|  | Total | 53 (21.8%) | | 118 (48.6%) | 49 (20.2%) | 23 (9.5%) | 243 (100%) |  |
| Stage | 0-Ⅱ | 33^a^ (20.8%) | | 77^a^ (48.4%) | 30^a^ (18.9%) | 19^a^ (11.9%) | 159 (100%) | 3.33 0.34 |
|  | Ⅲ-Ⅳ | 26^a^ (24.5%) | | 51^a^ (48.1%) | 23^a^ (21.7%) | 6^a^ (5.7%) | 106 (100%) |  |
|  | Total | 59 (22.3%) | | 128 (48.3%) | 53 (20.0%) | 25 (9.4%) | 265 (100%) |  |

Same-letter superscripts indicate no statistical difference (*p*＞0.05).

Table S6. Constituent ratios of Breslow thickness and stage in different marital status

| Factors | | Married | Other status | Total | chi-square test |
| --- | --- | --- | --- | --- | --- |
|  |  |  |  |  | **χ^2^ *p* value** |
| Breslow thickness | ≤2mm | 81^a^ (98.8%) | 1^a^ (1.2%) | 82 (100%) | 3.65 0.06 |
|  | ＞2mm | 150^a^ (93.2%) | 11^a^ (6.8%) | 161 (100%) |  |
|  | Total | 231 (95.1%) | 12 (4.9%) | 243 (100%) |  |
| Stage | 0-Ⅱ | 154^a^ (96.9%) | 5^a^ (3.1%) | 159 (100%) | 3.63 0.06 |
|  | Ⅲ-Ⅳ | 97^a^ (91.5%) | 9^a^ (8.5%) | 106 (100%) |  |
|  | Total | 251 (94.7%) | 14 (5.3%) | 265 (100%) |  |

Same-letter superscripts indicate no statistical difference (*p*＞0.05).
