# Supplementary figures and images for: Socioeconomic status correlates with clinical outcomes in patients with acral melanoma
Source: Front Public Health. 2025 Feb 3;13:1496082. doi: 10.3389/fpubh.2025.1496082 (PMC11830742; doi:10.3389/fpubh.2025.1496082)

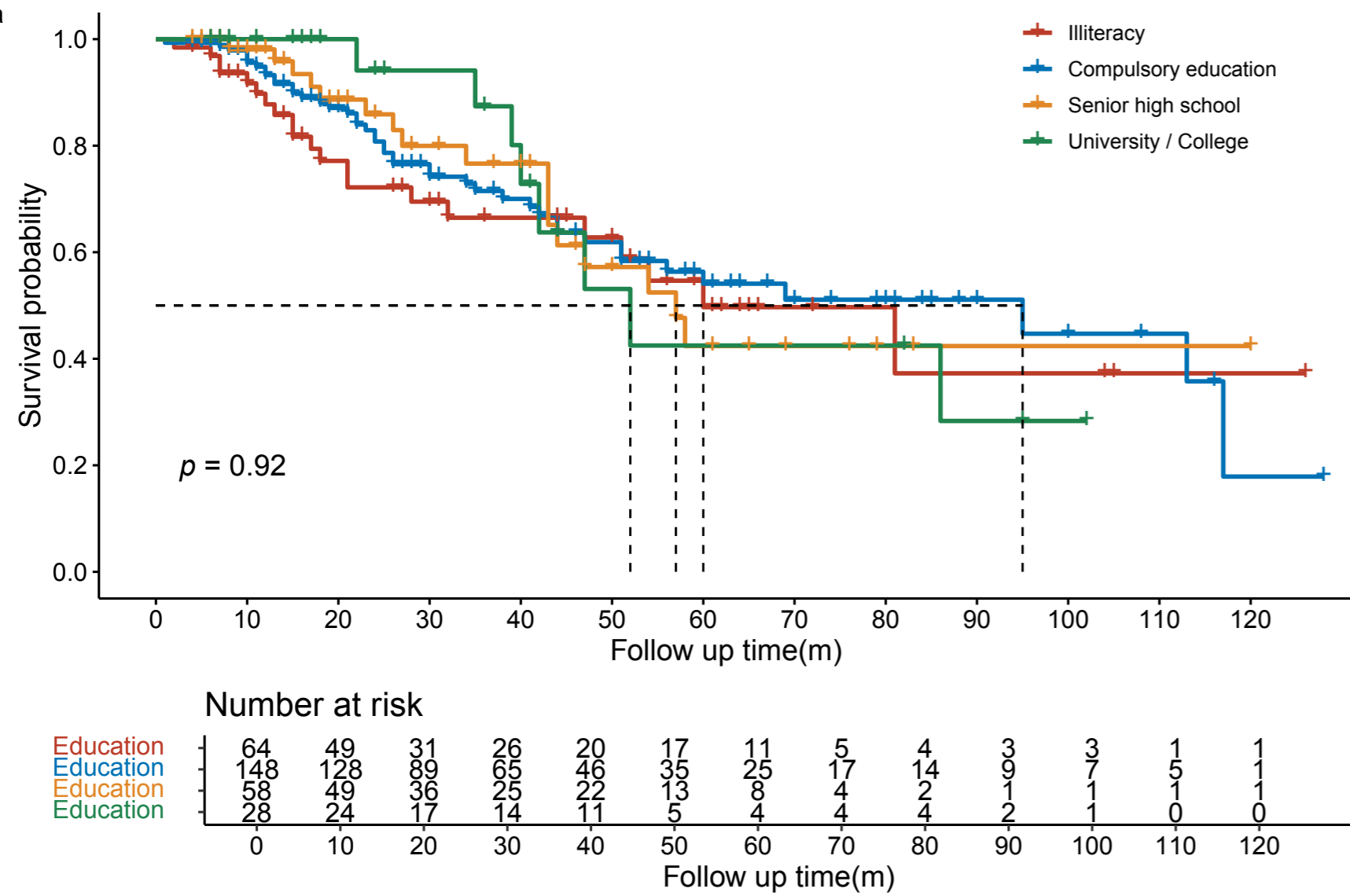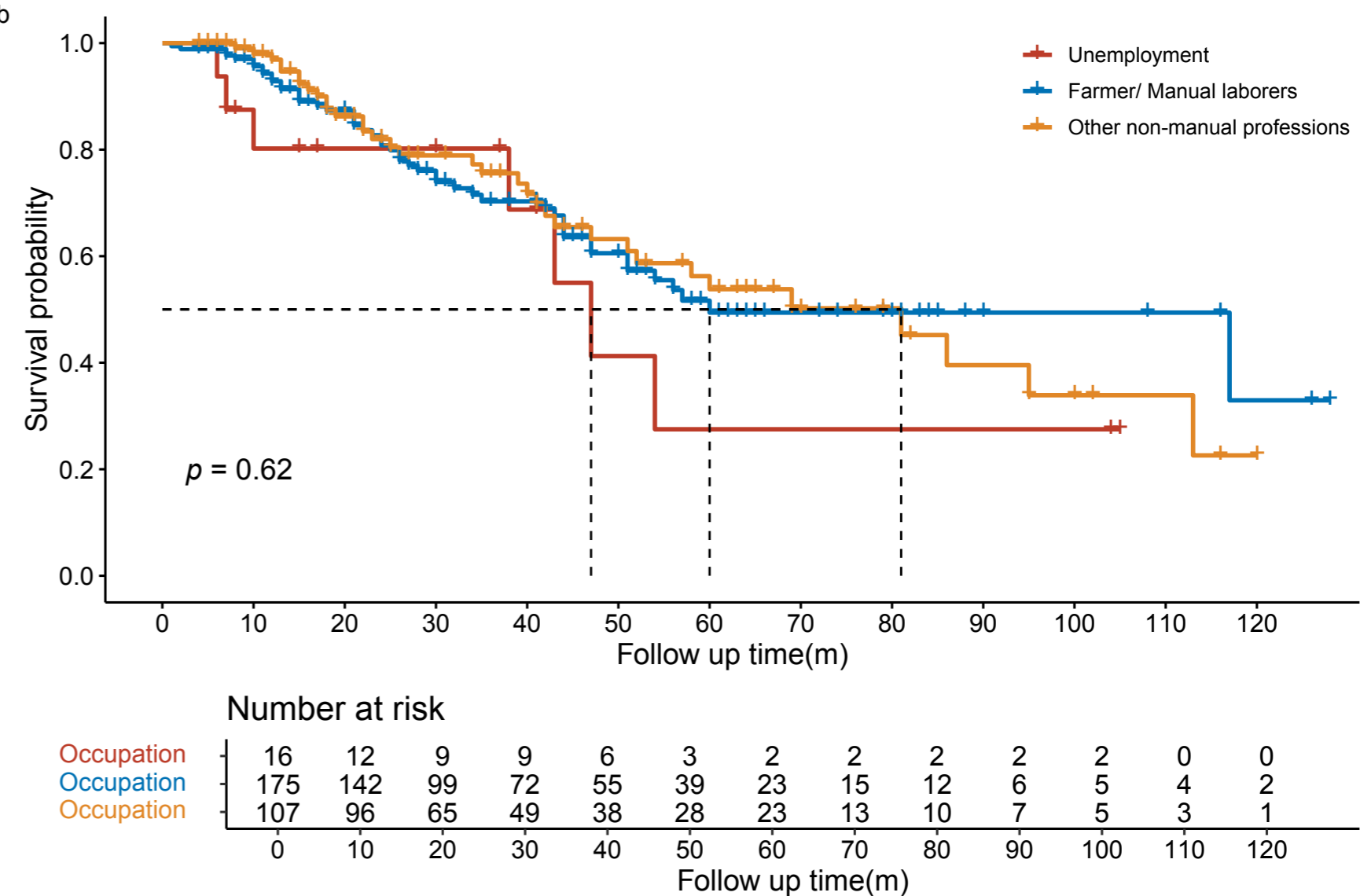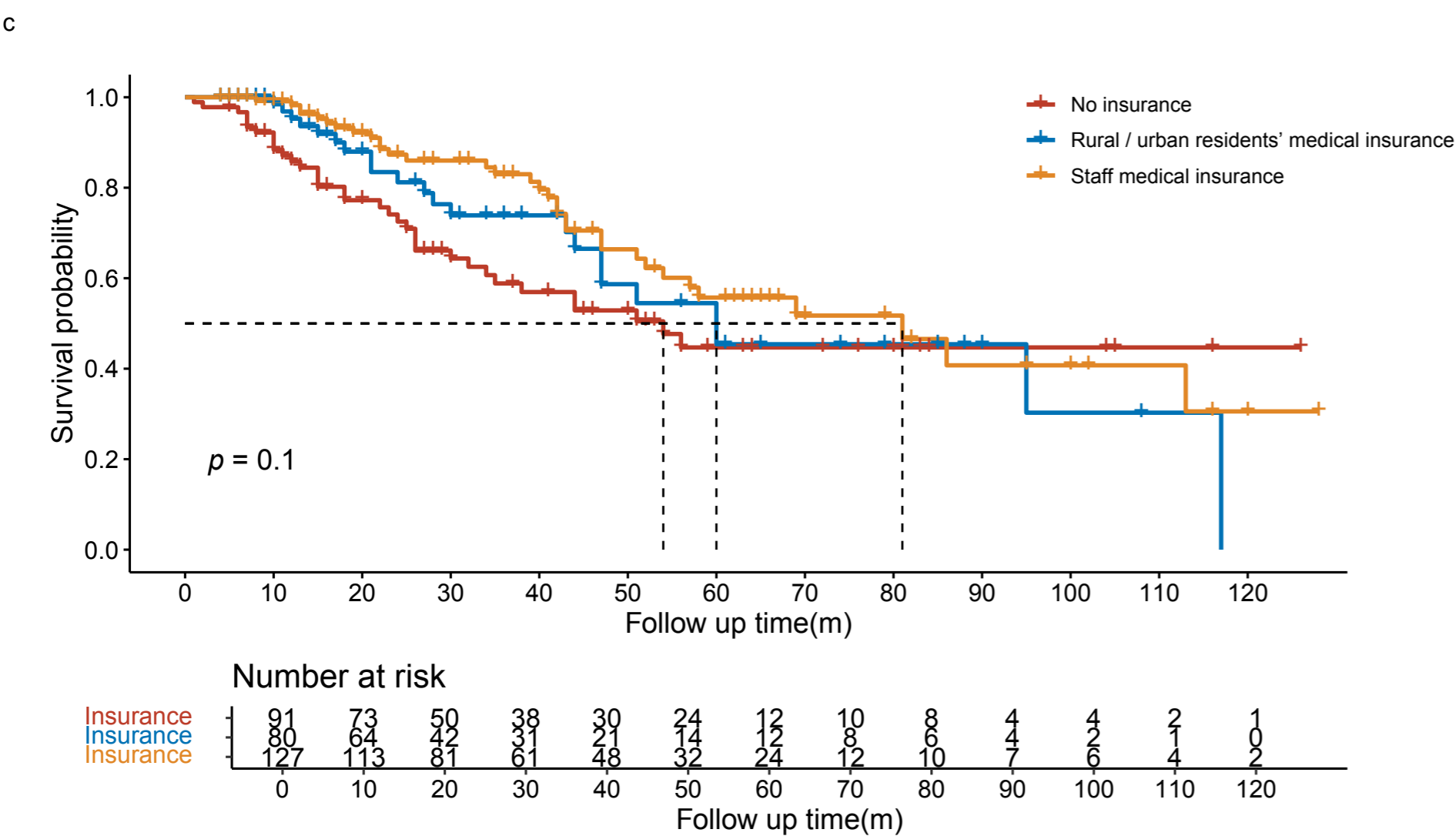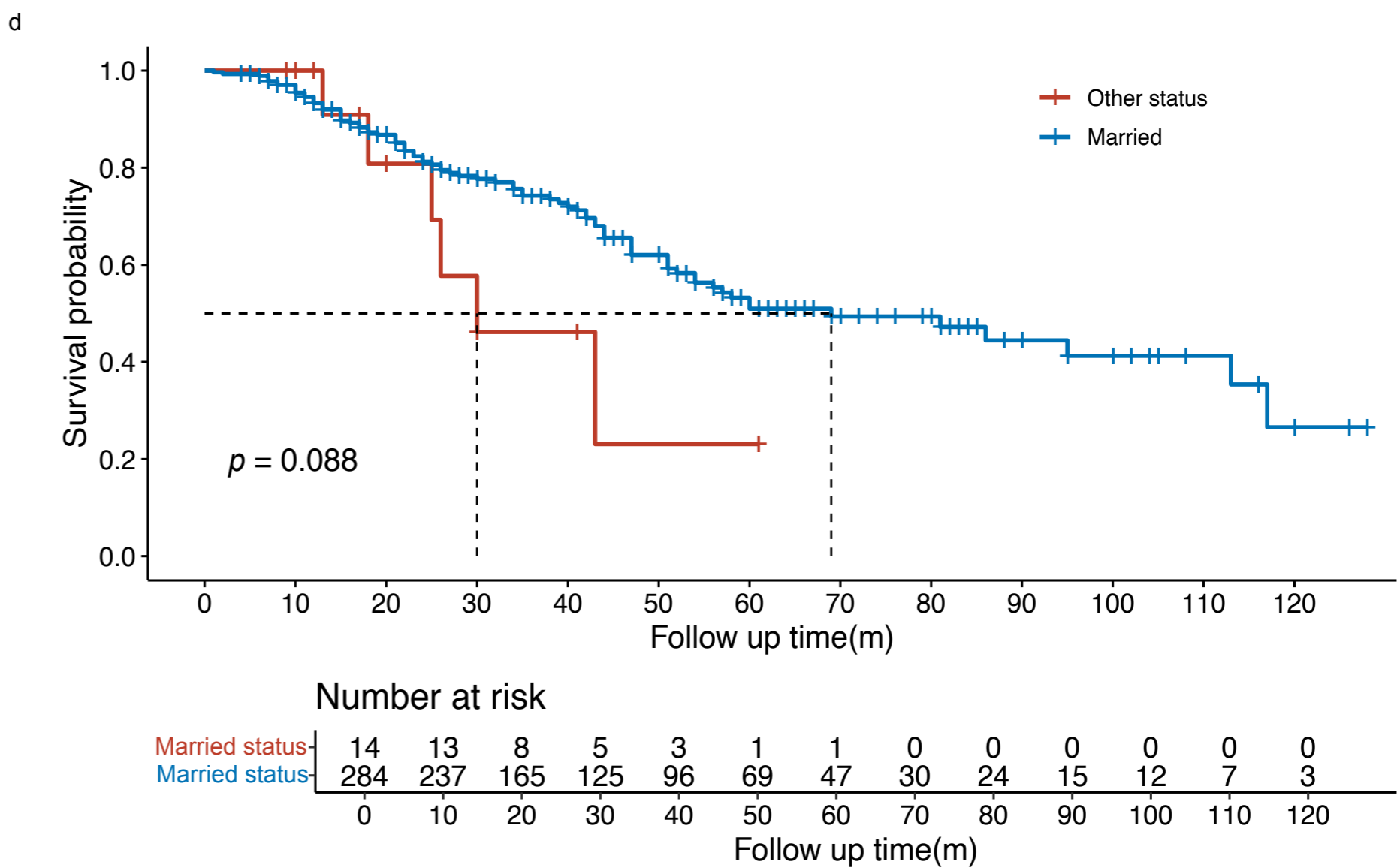

Supplement: Supplementary file 2 [file Data_Sheet_1.pdf]
